# Anxiety Symptoms in Preschool Children Born Very Preterm: Associations with Cognition and Neonatal Striatal Volumes

**DOI:** 10.3390/children13050695

**Published:** 2026-05-19

**Authors:** Carmen Rodríguez-Barrios, Natalia Jiménez-Luque, Yolanda Marín Almagro, Irene Gutierrez-Rosa, Manuel Lubián-Gutiérrez, Bahram Jafrasteh, Isabel Benavente-Fernández, Simón Pedro Lubián-López

**Affiliations:** 1 Department of Paediatrics, Puerta del Mar University Hospital, 11009 Cadiz, Spain; carmen.rodriguezbarrios@alum.uca.es (C.R.-B.);; 2 Biomedical Research and Innovation Institute of Cadiz (INiBICA), Research Unit, Puerta del Mar University Hospital, 11009 Cadiz, Spain

**Keywords:** very preterm birth, anxiety symptoms, neonatal magnetic resonance imaging, striatal volumes, cognitive outcome

## Abstract

**Highlights:**

**What are the main findings?**

• One in four very preterm preschoolers showed clinically significant anxiety, often alongside lower cognitive scores.
• Neonatal term-equivalent MRI showed that striatal volumetric organization, particularly the putamen–caudate interaction, was associated with later anxiety symptoms, independently of cognition.

**What are the implications of the main findings?**

• Emotional and cognitive follow-up should be considered together in children born very preterm, as anxiety symptoms are frequent and co-occur with poorer cognitive outcomes.
• Early neonatal MRI markers may help identify children at increased risk of later anxiety symptoms and support earlier monitoring and targeted intervention.

**Abstract:**

**Background/Objectives:** Children born very preterm (VP) are at increased risk of later emotional and cognitive difficulties, including anxiety symptoms during childhood. Altered early brain development, particularly within subcortical circuits involved in emotional regulation, may contribute to this vulnerability. This study aimed to assess anxiety symptoms in preschool-aged children born VP, examine their relationship with cognitive performance, and determine whether neonatal brain volumes at term-equivalent age (TEA) were associated with later anxiety symptoms. We also explored whether cognition mediated the association between neonatal striatal volumes and anxiety. **Methods:** We conducted a longitudinal cohort study of infants born at ≤32 weeks of gestation and/or with a birth weight ≤1500 g admitted to a tertiary neonatal intensive care unit between 2018 and 2021. At 4–6 years of age, anxiety symptoms were assessed using the Child Behavior Checklist (CBCL) Anxiety Problems subscale, and cognitive performance was evaluated with the Wechsler Preschool and Primary Scale of Intelligence-IV (WPPSI-IV). Neonatal magnetic resonance imaging performed at TEA was used to obtain regional brain volumetric measures. Associations were analyzed using adjusted linear regression, interaction-based volumetric modeling, path analysis, and receiver operating characteristic analysis. **Results:** Ninety-five children were included, and 24 (25.3%) showed clinically relevant anxiety symptoms according to the CBCL Anxiety Problems subscale. Higher WPPSI-IV scores were associated with lower anxiety scores (β = −0.183; *p* = 0.042). The best-fitting MRI model included caudate volume, putamen volume, and their interaction, with a significant association between the putamen–caudate interaction and anxiety symptoms (β = −17.807; *p* < 0.001). In the path model, both cognition and the putamen–caudate interaction were directly associated with anxiety, whereas the indirect effect through cognition was not significant. The final MRI model showed acceptable discrimination for clinically relevant anxiety (AUC = 0.796). **Conclusions:** Anxiety symptoms were frequent in preschool-aged children born VP and were associated with lower cognitive performance. Neonatal striatal volumetric organization, particularly the interaction between the putamen and caudate volumes, was independently associated with later anxiety symptoms, suggesting that cognitive and early neural factors may contribute to anxiety risk through parallel rather than mediated pathways.

## 1. Introduction

Advances in perinatal care have substantially improved the survival of very low birthweight (VLBW) and very preterm (VP) infants. Nevertheless, VP birth remains strongly associated with adverse long-term neurodevelopmental outcomes. This vulnerability is largely explained by the VP interruption of a critical period of brain maturation during the third trimester [1]. Consequently, the immature brain is left in a state of heightened vulnerability during neonatal intensive care hospitalization and is exposed to multiple insults, including white matter injury (WMI), germinal matrix-intraventricular hemorrhage (GMH-IVH), and impaired maturation. These processes may result in both overt structural lesions and more subtle but persistent alterations in brain development, which contribute to a wide spectrum of neurodevelopmental difficulties throughout infancy and childhood [2,3].

Anxiety symptoms are a particularly prevalent psychiatric complication among children born VP. A recent meta-analysis demonstrated that VP-born children have significantly greater odds of generalized anxiety disorder and specific phobias than their term-born peers. In line with this, the prevalence of clinically relevant anxiety problems in VP populations has been reported to range from 25% to 30% during early childhood [4]. Prematurity-related brain dysmaturation and neonatal brain injury may contribute to this increased vulnerability by altering the neural circuits involved in emotional regulation and fear processing [5].

Cognitive deficits are common among children born VP and are among the most consistent neurodevelopmental consequences of prematurity. These difficulties include impairments in executive functions, processing speed, working memory, and attention, and tend to persist across childhood and adolescence, with limited evidence of substantial catch-up over time [6,7,8,9]. Anxiety symptoms are also frequent in this population, and cognitive and emotional difficulties often coexist and may influence one another across development.

Reductions in brain volume, particularly in cortical and subcortical gray matter, have been increasingly associated with both anxiety symptoms and cognitive difficulties after VP birth. Structural neuroimaging studies of anxiety have consistently implicated prefrontal-limbic-striatal regions, with evidence supporting abnormalities in orbitofrontal and related regulatory areas [5,6,10]. In parallel, studies in VP and other neurodevelopmentally vulnerable populations have shown that smaller total brain, cortical gray matter, and white matter volumes are associated with poorer neurodevelopmental and cognitive outcomes [5,11]. Lower gestational age (GA) has also been linked to smaller cortical and subcortical volumes that partly mediate later deficits in cognition and school achievement [12]. Taken together, these findings suggest that volumetric brain alterations may contribute to both anxiety symptoms and cognitive impairment through disruption of neural circuits involved in emotional regulation, fear processing, and higher-order cognitive control. Within these networks, striatal development appears to be altered after prematurity. The caudate and putamen are key components of frontostriatal circuits involved in emotional regulation, salience processing, habit- and avoidance-related behavior, and cognitive control, and their altered development may contribute to both cognitive difficulties and anxiety symptoms in children born VP [13].

This study aimed to determine the prevalence of anxiety symptoms in preschoolers born very preterm, explore their relationship with cognitive performance, and assess whether neonatal striatal brain volumes predict later anxiety risk.

## 2. Materials and Methods

### 2.1. Study Population

This was a longitudinal cohort study of VP and/or VLBW infants admitted to the neonatal intensive care unit of Hospital Puerta del Mar between January 2018 and August 2021. Eligible infants were those with birth weight ≤ 1500 g and/or GA ≤ 32 weeks. Infants with congenital or chromosomal anomalies, metabolic disorders, central nervous system infections, or neonatal death were excluded. The study was approved by the Research Ethics Committee, and written informed consent was obtained from the parents or legal guardians of all participants.

The variables analyzed included prenatal variables (multiple pregnancy, in vitro fertilization, preeclampsia, gestational diabetes, chorioamnionitis, maternal education level, maternal age); perinatal variables (GA at birth, sex, birth weight, type of delivery); and postnatal variables (bronchopulmonary dysplasia (BPD), hemodynamically significant persistent ductus arteriosus (HSPDA), late sepsis, necrotizing enterocolitis, retinopathy of prematurity (ROP) and days of mechanical ventilation). The presence of neurological injury was assessed using magnetic resonance imaging (MRI) at term-corrected GA. Neonatal brain injury was assessed using MRI at TEA. MRI findings were classified according to the scoring system proposed by Kidokoro et al. [14], as previously applied in similar neonatal neuroimaging studies from our group [15]. Kidokoro score grades the development and lesions of the cortical and deep gray matter, white matter, and cerebellum. The overall score obtained classifies the MRI findings as follows: normal (0–3 points); or abnormal, which can be divided into mild (4–7 points), moderate (8–11 points), and severe (≥12 points). Children with a score of less than 8 points were considered to have normal MRI findings or findings compatible with mild involvement, whereas children with a score equal to or greater than 8 points had findings suggestive of moderate or severe impairment.

The GMH-IVH was graded according to the classification proposed by Volpe [16]. Grades I and II GMH-IVH were considered mild, whereas grade III and PHI were considered moderate to severe GMH-IVH.

### 2.2. Brain Magnetic Resonance Imaging

MRI was performed using a 1.5 T Magneton Symphony scanner (Siemens Health Care, Erlangen, Germany) located in the radiology unit. T1-weighted images were obtained using a three-dimensional spoiled gradient [repetition time 1660 (RT)/echo time 5.16 (ET)] and transverse T2-weighted images with turbo spin echo (4180.00/98.00).

Segmentation of regional brain volumes for the structures of interest was performed by multi-atlas segmentation using MELAGE V1, a software platform for segmentation, visualization, and semi-manual correction developed by our group [17,18]. For bilateral structures, averaged regional volumes were used in the analyses.

The segmentation of the brain structures in T1-weighted images involved a systematic approach that enhances accuracy and precision. The process begins with brain extraction, using a specialized image reconstruction and brain extraction tool. Following extraction, brain tissue was segmented using an enhanced spatial fuzzy C-means algorithm for 3D T1 MRI segmentation [18]. The resulting tissue segmentation maps, particularly those from the gray matter region, are then subsequently employed to register each T1 image to the MCRIB 2.0 atlas [19]. This registration process aligns the individual images with a standardized anatomic framework, facilitating consistent comparisons across patients. Once registration is complete, label information from the MCRIB 2.0 atlas is assigned to various regions of interest in the T1 images. This step is pivotal because it provides a comprehensive mapping of the anatomic structures within the images. The resultant segmentation was validated using the MELAGE software and by specialists in our group.

### 2.3. Neurodevelopment and Behavioral Assessment

Cognitive performance was assessed using the Wechsler Preschool and Primary Scale of Intelligence-IV Edition (WPPSI-IV). The primary cognitive outcome was total Intelligence Quotient (IQ). As a secondary/sensitivity outcome, poor cognitive outcome was defined as total IQ lower than 85. Cognitive assessments were conducted by trained psychologists blinded to the neonatal MRI findings.

Behavioral outcome was assessed using the Child Behavior Checklist (CBCL). For the present analysis, we focused on the Anxiety Problems subscale, given the high frequency of anxiety symptoms among children born VP. Both the continuous T-score and a dichotomized clinically relevant outcome were examined. The dichotomized Anxiety Problems score variable was defined as a T-score over 65.

### 2.4. Statistical Analysis

Categorical variables were summarized as frequencies and percentages, whereas continuous variables were summarized as mean ± standard deviation (SD) or median [interquartile range, IQR], as appropriate according to their distribution. The main behavioral outcome was the CBCL Anxiety Problems score, analyzed both as a continuous T-score and as a dichotomized clinically relevant outcome, defined as a T-score > 65.

The association between WPPSI-IV IQ score and the score obtained in the Anxiety Problems subscale was assessed using linear regression. Models were adjusted for GA at birth and chronological age at assessment.

To identify the MRI model most strongly associated with anxiety symptoms, an exhaustive search of pairwise volumetric interaction models was performed using TEA-MRI measures, using a hierarchical approach in which GA at birth and PMA at TEA-MRI were included in all models. Model performance was ranked according to adjusted R^2^, the Akaike information criterion, and the Bayesian information criterion, and the 10 best-fitting models were reviewed, before the final model was selected based on overall model fit, consistency across selection criteria, and biological interpretability.

The selected MRI model was then incorporated into an observed-variable path model. Direct, indirect, and total effects were estimated using robust standard errors.

For the dichotomized anxiety outcome, the discriminative performance of the final MRI model was assessed using logistic regression and receiver operating characteristic analysis. The area under the curve was calculated, and the optimal probability cutoff was determined using the Youden index, with the corresponding sensitivity and specificity.

Statistical analysis was performed using Stata 16.0 (Stata Statistical Software: Release 16. College Station, TX, USA: StataCorpLP). Statistical significance was defined as a two-sided *p* value < 0.05.

## 3. Results

### 3.1. Study Population

**Figure 1:**
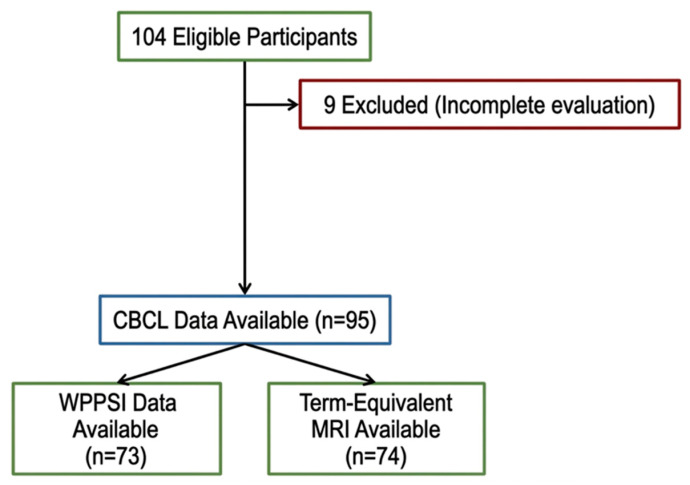
Flow diagram of study population. CBCL: Child Behavior Checklist; WPPSI: Wechsler Preschool and Primary Scale of Intelligence; MRI: Magnetic Resonance Imaging.

Among 104 children born VP who were eligible for follow-up assessment at 4–6 years of age, 9 did not complete the full assessment; therefore, 95 were included in the final study population. Mean GA at birth was 29.4 ± 2.1 weeks, 49 (52.7%) were female, and mean birth weight was 1191.6 ± 350.8 g. Among infants with available neuroimaging data, moderate-to-severe MRI abnormalities based on the Kidokoro score were identified in 9 patients, and grade 3 GMH-IVH and/or PHI was present in 7 patients (The other variables are described in detail in Table 1, and the study flowchart is presented in Figure 1).

Clinically relevant CBCL scores for anxiety were identified in 24/95 (25.3%). Mean anxiety score was 59.25 (±8.148). WPPSI assessments were available in 73 children, of whom 13 had a score below 85, consistent with poor cognitive outcome. Analyses involving cognitive performance were therefore restricted to participants with available WPPSI-IV data.

### 3.2. Associations of Anxiety Problems with Cognitive Outcomes

In adjusted linear regression models including GA at birth and chronological age at assessment, higher WPPSI scores were associated with lower anxiety scores in CBCL evaluation (β = −0.183; 95% CI −0.359 to −0.007; *p* = 0.042).

### 3.3. Selection of Term-Equivalent MRI Volumetric Interaction Models Associated with Anxiety Problems

**Figure 2:**
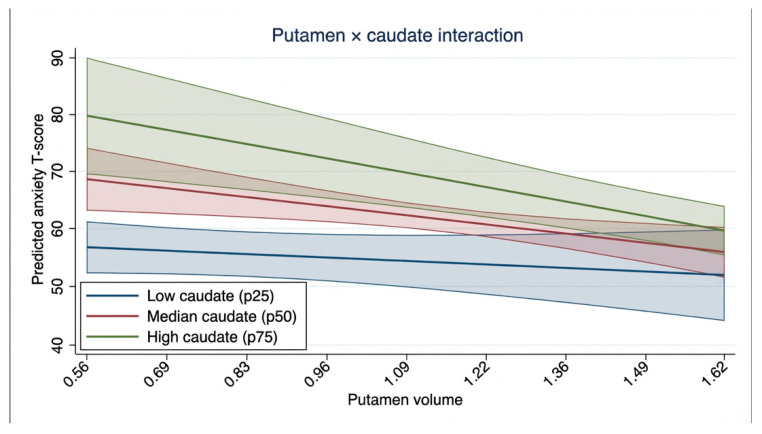
Predicted anxiety score according to putamen volume at low (p25), median (p50), and high (p75) caudate volumes.

To identify the MRI model most strongly associated with anxiety symptoms, the top 10 pairwise MRI volumetric interaction models derived from an exhaustive model search and ranked according to goodness-of-fit metrics, were then reviewed (Supplementary Material: Table S1). GA at birth and PMA at TEA-MRI were retained in all models. Among the models, the best-fitting model included caudate volume, putamen volume, and their interaction. In this model, caudate volume was associated with anxiety score (β = 38.29, *p* < 0.001), whereas putamen volume showed no association (β = 5.92, *p* = 0.374). The putamen and caudate interaction was associated with anxiety problems (β = −17.81, *p* < 0.001), indicating that the positive association between caudate volume and anxiety score became weaker at higher putamen volumes (Table 2). Adjusted predictive margins showed a steeper decline in predicted anxiety score across increasing putamen volume at higher caudate volumes (Figure 2).

### 3.4. Path Model Linking Anxiety, Putamen-Caudate Interaction, and Cognitive Outcome

**Figure 3:**
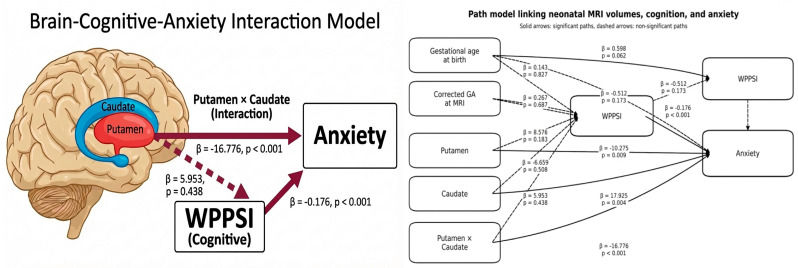
Observed-variable path model examining direct relationships between gestational age at birth, postmenstrual age at term-equivalent MRI, putamen volume, caudate volume, their interaction, cognitive outcome (WPPSI), and anxiety symptoms.

Given the observed association between anxiety symptoms and cognitive outcome, we next used a path model to explore the relationship between the selected MRI interaction model, WPPSI, and anxiety within a single framework (Supplementary Material: Tables S2–S5). The model included GA at birth, PMA at MRI, putamen volume, caudate volume, their interaction, WPPSI score, and anxiety score. In this model, higher WPPSI scores were associated with lower anxiety scores (β = −0.176, *p* < 0.001). The putamen and caudate interaction remained directly associated with anxiety symptoms (β = −16.776, *p* < 0.001), as did putamen volume (β = −10.275, *p* = 0.009) and caudate volume (β = 17.925, *p* = 0.004) (Figure 3). However, putamen, caudate, and their interaction were not significantly associated with WPPSI, and the indirect effect of the putamen and caudate interaction through cognition was not significant (β = −1.046, *p* = 0.485) (Table 3). These findings indicate that cognitive outcome and the putamen–caudate interaction were both associated with anxiety, but through parallel rather than mediated pathways.

**Figure 4:**
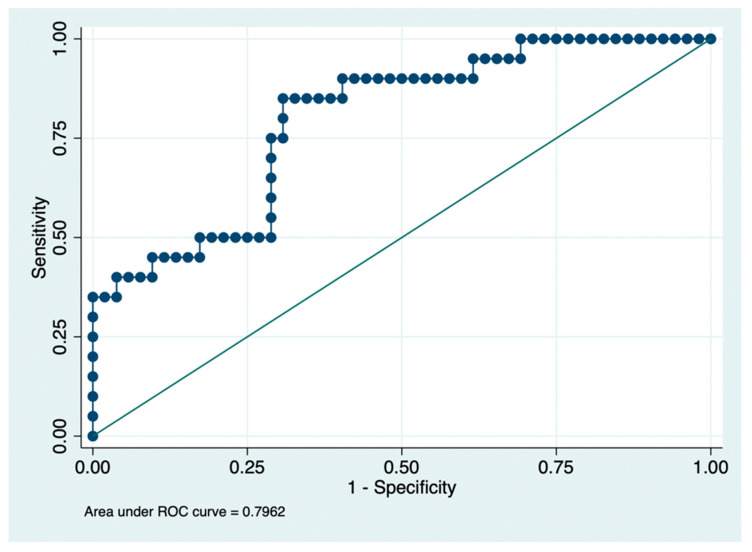
Receiver operating characteristic (ROC) curve for the logistic model predicting clinically relevant anxiety status from gestational age at birth, postmenstrual age at term-equivalent MRI, putamen volume, caudate volume, and their interaction.

To explore the discriminative performance of this MRI model for clinically relevant anxiety status, a logistic regression model including GA, PMA at MRI, putamen volume, caudate volume, and their interaction was evaluated with ROC analysis, which demonstrated acceptable-to-good discrimination, with an area under the curve of 0.80 (Figure 4). The optimal probability cutoff according to the Youden index was 0.23, corresponding to a sensitivity of 85.0% and a specificity of 69.2% (Table 4). These findings indicate that the putamen–caudate model had moderate discriminative ability to identify children with clinically relevant anxiety symptoms.

## 4. Discussion

In this cohort of preschool-aged children born VP, clinically relevant anxiety symptoms were present in 25.3% of the population. Anxiety scores were inversely associated with cognitive performance, and neonatal MRI findings further suggest that early striatal organization was related to later anxiety symptoms. Specifically, the best-fitting volumetric model included caudate volume, putamen volume, and their interaction, and this association remained robust in the path model. Notably, however, the relationship between the putamen–caudate interaction and anxiety was not mediated by cognition. Together, these findings suggest that anxiety after VP birth may reflect both a broader neurodevelopmental vulnerability and a more specific alteration in early subcortical brain development.

The proportion of children with clinically relevant anxiety symptoms in our cohort underscores the clinical importance of emotional outcomes after VP birth. The inverse association between WPPSI and anxiety scores indicates that emotional and cognitive difficulties tend to co-occur in this population. This pattern is consistent with the concept that prematurity-related disturbances in brain maturation may give rise to a multidimensional phenotype in which cognitive and emotional outcomes are linked, although not necessarily through the same mechanisms [20,21].

The MRI findings provide a more specific neurodevelopmental signal that may help contextualize later anxiety vulnerability. The selected model identified an interaction between putamen and caudate volumes, suggesting that the relative organization of striatal structures may be more informative than isolated regional measures. This is biologically plausible, given the central role of the striatum in emotional regulation, motivation, and decision-making, and the known vulnerability of these subcortical regions to prematurity-related dysmaturation [22,23]. The observed interaction, whereby the association between caudate volume and anxiety was attenuated at higher putamen volumes, raises the possibility that preserved putamen development may partly modulate the behavioral effects of caudate alteration. These findings are consistent with the relevance of coordinated cortico-striatal–limbic development for later anxiety vulnerability after VP birth.

The path analysis offers further insight into the relationships observed. Although lower WPPSI scores were associated with higher anxiety, neither putamen volume, caudate volume, nor their interaction was significantly associated with WPPSI, and the indirect effect through cognition was not significant. These findings do not support a model in which the association between neonatal striatal development and anxiety is explained by global cognitive ability. Instead, cognition and neonatal striatal organization may contribute to later anxiety through partly distinct pathways.

The final MRI model showed acceptable discrimination for clinically relevant anxiety symptoms. The combination of GA, PMA at MRI, putamen volume, caudate volume, and their interaction yielded an AUC of 0.80, with high sensitivity and moderate specificity. Although these estimates should be interpreted cautiously given the sample size, they are consistent with prior studies showing that early deep gray matter development in VP infants is associated with later neurodevelopmental outcome. In particular, longitudinal growth of the caudate and the putamen have been related to later cognitive performance, suggesting that neonatal deep gray matter measures may capture clinically relevant variation in early brain development [24].

From a clinical perspective, these findings suggest that follow-up programs for children born VP should not focus exclusively on cognitive and motor outcomes, but should also include systematic emotional screening. In particular, the co-occurrence of anxiety symptoms and lower cognitive performance supports an integrated approach in which emotional and cognitive development are monitored together. However, this predictive model should be interpreted as exploratory. External validation in independent and larger cohorts is required before these MRI-based markers can be considered for clinical risk stratification or implementation in follow-up programs.

Several limitations should be acknowledged. The sample size was modest, particularly for analyses integrating MRI, cognition, and behavior, which may have reduced precision and increased susceptibility to model instability. Although the number of children lost to follow-up was relatively small, selection bias cannot be excluded, as children lost to follow-up may have differed systematically from those included in the final sample. The single-center design and the inclusion of a Spanish cohort may limit the generalizability of the findings to other populations A limitation is the reliance on parent-reported CBCL scores, which may not capture clinically diagnosed anxiety disorders. Future studies should include standardized clinical interviews. Although the analyses accounted for key maturational variables, residual confounding variables related to neonatal morbidity, family context, and later environmental exposures cannot be excluded. In addition, the MRI analysis was restricted to volumetric measures at TEA and did not capture subsequent developmental trajectories, microstructural alterations, or functional connectivity.

This study has several strengths. It combines neonatal TEA-MRI, preschool cognitive assessment, and behavioral evaluation in the same cohort of children born VP. The use of quantitative volumetric measures and interaction-based modeling allowed assessment of coordinated regional development rather than isolated brain volumes alone. The inclusion of path analysis also enabled examination of whether cognition accounted for the observed brain–behavior associations. Finally, the analysis of discriminative performance for clinically relevant anxiety increases the potential clinical relevance of the findings.

## 5. Conclusions

Anxiety symptoms were frequent in this cohort of children born VP and were associated with lower cognitive performance at preschool age. Neonatal striatal volumetric organization, particularly the interaction between putamen and caudate volumes, was also associated with later anxiety symptoms. However, this brain–anxiety relationship was not mediated by cognition, suggesting that cognitive and neural contributions to anxiety may operate through parallel rather than sequential pathways. These findings support the relevance of early brain development for later emotional outcomes after VP birth and highlight the potential value of neonatal MRI markers in identifying children at risk of anxiety symptoms. Future research should evaluate these findings in larger multicenter cohorts and incorporate longitudinal follow-up and multimodal neuroimaging to clarify the developmental pathways linking early striatal organization with later anxiety symptoms.

## Figures and Tables

**Table 1:** Perinatal characteristics of patients who underwent assessment using CBCL. Continuous variables are presented as mean ± SD or median [IQR], and categorical variables as n/N (%). GA: gestational age; SGA: small for gestational age; IVF: in vitro fertilization; BPD: bronchopulmonary dysplasia; HSPDA: hemodynamic significant persistent ductus arteriosus; NEC: necrotizing enterocolitis; ROP: retinopathy of prematurity; GMH-IVH: germinal matrix -intraventricular hemorrhage; PHI: periventricular hemorrhagic infarction; MV: mechanical ventilation.

		N = 95	
GA at birth (weeks)		29.427 (±2.169)	
Sex (F)		49 (52.69%)	
Birth weight (g)		1191.559 (±350.749)	
SGA		N = 93	13 (13.98%)
Maternal age at delivery (years)		N = 93	33.193 (±5.724)
Chronological age at assessment (years)		4.753 (±0.591)	
Cesarean section		60 (63.16%)	
Prenatal steroids		N = 94	78 (82.98%)
Chorioamnionitis		N = 77	21 (27.27%)
Multiple pregnancy		N = 93	32 (34.41%)
IVF		N = 93	15 (16.13%)
Preeclampsia		N = 86	17 (19.77%)
Diabetes		8 (8.51%)	
Mothers’ level of education (N = 92)	Basic	69 (75%)	
	Intermediate	19 (20.65%)	
	Advanced	4 (4.35%)	
BPD (mod-severe)		N = 72	11 (14.86%)
HSPDA		N = 72	8 (11.11%)
Late onset sepsis		16 (16.84%)	
Severe NEC		1 (1.05%)	
ROP (mod-severe)		N = 71	3 (4.23%)
Brain injury as Kidokoro score (mod-severe)		N = 69	9 (13.04%)
GMH-IVH	No	53 (71.62%)	
	Grade 1	10 (13.51%)	
	Grade 2	4 (5.41%)	
	Grade 3	4 (5.41%)	
	PHI	3 (4.05%)	
GMH 3 and/or PHI		N = 70	7 (10%)

**Table 2:** The linear regression model including putamen volume, caudate volume, and their interaction. The model was adjusted for GA at birth and PMA at term-equivalent MRI; these coefficients, as well as the constant, are omitted from the main table for brevity. The adjustment coefficients were as follows: GA at birth, β = 0.572, 95% CI −0.171 to 1.314, *p* = 0.129; PMA at term-equivalent MRI, β = −0.561, 95% CI −1.356 to 0.235, *p* = 0.164; constant, β = 41.422, 95% CI 10.124 to 72.721, *p* = 0.010. GA: gestational age; PMA: postmenstrual age.

Variable	β	95% CI	*p*-Value
Putamen volume	5.924	−4.855 to 16.703	0.374
Caudate volume	38.294	20.745 to 55.844	<0.001
Putamen × Caudate	−17.807	−27.576 to −8.038	<0.001
N = 74		R^2^/Adj. R^2^ = 0.346/0.294	
F-statistic = 6.66		Model *p*-value = <0.001	

**Table 3:** Summary of SEM model linking neonatal MRI volumes, WPPSI, and anxiety. WPPSI: Wechsler Preschool and Primary Scale of Intelligence.

Effect Type	Path	β	*p*-Value
Direct path	Putamen × Caudate → WPPSI	5.953	0.438
Direct path	WPPSI → Anxiety	−0.176	<0.001
Direct path	Putamen × Caudate → Anxiety	−16.776	<0.001
Indirect effect	Putamen × Caudate → WPPSI → Anxiety	−1.046	0.485
Total effect	Putamen × Caudate → Anxiety	−17.823	<0.001

**Table 4:** Receiver operating characteristic (ROC) performance of the logistic model predicting clinically relevant anxiety status from gestational age at birth, postmenstrual age at term-equivalent MRI, putamen volume, caudate volume, and their interaction. AUC: area under the curve.

Outcome	AUC	Optimal Cutoff	Sensitivity	Specificity	Youden Index
Anxiety	0.796	0.226	0.850	0.692	0.542

## Data Availability

The original contributions presented in this study are included in the article. Further inquiries can be directed to the corresponding author.

## Supplementary Materials

The following supporting information can be downloaded at https://www.mdpi.com/article/10.3390/children13050695/s1. Table S1: Top 10 candidate interaction models for anxiety T-score. GA: gestational age; PMA: postmenstrual age. Table S2: Model fit summary. Table S3: Direct effects. Table S4: Indirect effects. Table S5: Total effects on anxiety.

## Abbreviations

The following abbreviations are used in this manuscript:

VP	Very Preterm
VLBW	Very low birth weight
CBCL	Child Behavior Checklist
WPPSI-IV	Wechsler Preschool and Primary Scale of Intelligence-IV
