# Supplementary material for: Anxiety Symptoms in Preschool Children Born Very Preterm: Associations with Cognition and Neonatal Striatal Volumes
Source: Children (Basel). 2026 May 19;13(5):695. doi: 10.3390/children13050695 (PMC13204709; doi:10.3390/children13050695)
Supplement: Supplementary file 1 [file children-13-00695-s001.zip › children-4301418-supplementary.pdf]

Anxiety Model Selection Tables

Supplementary Table S1. Top 10 candidate interaction models for anxiety T-score. GA: Gestational Age; PMA:postmenstrual age.

Rank	Interaction term	Adj. R^2	AIC	BIC	Interaction β	p-value
1	GA+ PMA+ Putamen × Caudate	0.294	463.0	476.4	-17.807	<0.001
2	GA+ PMA+ Putamen × Cingulate	0.211	470.6	484.0	-20.432	0.002
3	GA+ PMA+ Putamen × Amygdala	0.208	470.9	484.3	-37.585	0.012
4	GA+ PMA+ Thalamus × Putamen	0.207	471.0	484.4	-8.668	0.004
5	GA+ PMA+ Putamen × Hippocampus	0.204	471.3	484.7	-23.532	0.004
6	GA+ PMA+ Putamen × Nucleus accumbens	0.189	472.6	486.0	-92.489	0.016
7	GA+ PMA+ Gray matter × Putamen	0.187	472.7	486.1	-0.306	0.006
8	GA+ PMA+ Pallidum × Caudate	0.184	473.0	486.4	-53.492	0.001
9	GA+ PMA+ White matter × Putamen	0.177	473.5	486.9	-0.282	0.022
10	GA+ PMA+ Gray matter × White matter	0.148	475.9	489.3	-0.003	0.051

SEM Path Model: Neonatal MRI Volumes, WPPSI, and Anxiety

Supplementary Table S2. Model fit summary

Model summary	Value
Estimation method	ML with robust standard errors
Log pseudolikelihood	-802.753
SRMR	0.000
Coefficient of determination (CD)	0.382
Main significant path	WPPSI → Anxiety (β = -0.176, p < 0.001)

Supplementary Table S3. Direct effects

Path	Estimate / SE / p	95% CI
GA → WPPSI	β 0.143<br>SE 0.652<br>p 0.827	-1.136 to 1.421
PMA → WPPSI	β 0.267<br>SE 0.663<br>p 0.687	-1.032 to 1.567
Putamen → WPPSI	β 8.576<br>SE 6.436<br>p 0.183	-4.038 to 21.190
Caudate → WPPSI	β -6.659<br>SE 10.067<br>p 0.508	-26.390 to 13.072
Putamen × Caudate → WPPSI	β 5.953<br>SE 7.671<br>p 0.438	-9.083 to 20.988
WPPSI → Anxiety	β -0.176<br>SE 0.047<br>p <0.001	-0.268 to -0.084
GA → Anxiety	β 0.598<br>SE 0.320<br>p 0.062	-0.029 to 1.225
PMA → Anxiety	β -0.512<br>SE 0.375<br>p 0.173	-1.248 to 0.224
Putamen → Anxiety	β -10.275<br>SE 3.950<br>p 0.009	-18.016 to -2.534
Caudate → Anxiety	β 17.925<br>SE 6.288<br>p 0.004	5.601 to 30.248
Putamen × Caudate → Anxiety	β -16.776<br>SE 3.744<br>p <0.001	-24.115 to -9.437

Supplementary Table S4. Indirect effects

Indirect path to Anxiety via WPPSI	Estimate / SE / p	95% CI
GA → WPPSI → Anxiety	β -0.025<br>SE 0.112<br>p 0.823	-0.244 to 0.194
PMA → WPPSI → Anxiety	β -0.047<br>SE 0.121<br>p 0.698	-0.284 to 0.190
Putamen → WPPSI → Anxiety	β -1.508<br>SE 1.111<br>p 0.175	-3.685 to 0.670
Caudate → WPPSI → Anxiety	β 1.171<br>SE 1.838<br>p 0.524	-2.433 to 4.774
Putamen × Caudate → WPPSI → Anxiety	β -1.046<br>SE 1.498<br>p 0.485	-3.982 to 1.889

Supplementary Table S5. Total effects on anxiety

Total effect on Anxiety	Estimate / SE / p	95% CI
GA	β 0.573<br>SE 0.335<br>p 0.087	-0.083 to 1.229
PMA	β -0.559<br>SE 0.355<br>p 0.116	-1.256 to 0.138
Putamen	β -11.783<br>SE 4.041<br>p 0.004	-19.702 to -3.863
Caudate	β 19.095<br>SE 5.839<br>p 0.001	7.651 to 30.540
Putamen × Caudate	β -17.823<br>SE 3.891<br>p <0.001	-25.449 to -10.196

WPPSI, Wechsler Preschool and Primary Scale of Intelligence; SRMR, standardized root mean squared residual; CD, coefficient of determination; GA: gestational age; PMA: postmenstrual ages
